# Infusion line contamination in preterm neonates: impact of infusion line design, length, and use duration: the multicenter ChronoBIOline study

**DOI:** 10.3389/fmicb.2024.1495568

**Published:** 2025-01-24

**Authors:** Sandra Dos Santos, Anne-Sophie Valentin, Mathilde Farizon, Manon Charbonneau, Mohamed Riadh Boukhris, Roselyne Brat, Fabiana Cazzorla, Jennifer Chauvel, Fabrice Cneude, Pauline Coutable, Maryvonne Demasure, Emeline Duminil, Vénonique Faraut-Derouin, Maud Gits Muselli, Valérie Gorin, Rosemary Goujon, Melinda Guillouche-Puissant, Nadine Hacinlioglu, Caroline Landelle, Annick Lefebvre, Elise Leroy-Terquem, Aurore Martinet, Camille Massebeuf, Nadia Mazille Orfanos, Guillaume Menard, Laure Menvielle, Vanessa Monin, Virginie Morange, Juliana Patkai, Nathalie Perrault, Emilie Prat, Nathalie van der Mee-Marquet

**Affiliations:** ^1^Centre Hospitalier Régional Universitaire, Hôpital Bretonneau, Mission Nationale Surveillance et Prévention des Infections Associées aux Dispositifs Invasifs, Centre d’Appui pour la Prévention des Infections Associées aux Soins en Région Centre val de Loire, Tours, France; ^2^Centre Hospitalier Universitaire, Service de Réanimation Néonatale, Lille, France; ^3^Centre Hospitalier Universitaire, Service de Réanimation Néonatale, Orléans, France; ^4^Hospital Hygiene Unit, Centre Hospitalier Universitaire Grenoble Alpes, Université Grenoble Alpes, CNRS, Grenoble INP, TIMC, Grenoble, France; ^5^Service de Néonatalogie, Centre Hospitalier, Saint Brieuc, France; ^6^Neonatal Intensive Care Unit, Centre Hospitalier Universitaire Grenoble Alpes, Grenoble, France; ^7^Service de Réanimation Néonatale, Centre Hospitalier Régional Universitaire, Tours, France; ^8^Service de Prévention du Risque Infectieux, Centre Hospitalier Universitaire, Orléans, France; ^9^Equipe Opérationnelle d’Hygiène, Centre Hospitalier, Calais, France; ^10^Equipe Opérationnelle d’Hygiène, Assistance Publique – Hôpitaux de Paris, Hôpital Cochin, Paris, France; ^11^Equipe Opérationnelle d’Hygiène, Assistance Publique – Hôpitaux de Paris, Hôpital Robert Debré, Paris, France; ^12^Service de Réanimation Néonatale, Hôpital Sud, Centre Hospitalier Universitaire, Rennes, France; ^13^Equipe Opérationnelle d’Hygiène, Centre Hospitalier Régional Universitaire, Reims, France; ^14^Service de Réanimation Néonatale, Assistance Publique – Hôpitaux de Paris, Hôpital Necker, Paris, France; ^15^Service de Bactériologie Hygiène-Hospitalière, UMR_S 1230, Centre Hospitalier Universitaire, Rennes, France; ^16^Service de Reanimation Néonatale et Pédiatrique, Centre Hospitalier Régional Universitaire, Reims, France; ^17^Service de Réanimation Néonatale, Assistance Publique – Hôpitaux de Paris, Hôpital Béclère, Clamart, France; ^18^Equipe Opérationnelle d’Hygiène, Centre Hospitalier Régional Universitaire, Tours, France; ^19^Service de Reanimation Néonatale, Assistance Publique – Hôpitaux de Paris, Hôpital Port-Royal, Paris, France; ^20^Service de Réanimation Néonatale, Assistance Publique – Hôpitaux de Paris, Hôpital Cochin, Paris, France; ^21^Unité de Prévention et de Contrôle de l’Infection, Centre Hospitalier, Saint Brieuc, France

**Keywords:** neonatalogy, infusion line, central line associated bacteremia, central venous catheter, umbilical catheter, peripherally inserted central catheter, *Staphylococcus haemolyticus*

## Abstract

**Introduction:**

Central venous catheters are critical in preterm neonatal care but increase the risk of central line-associated bloodstream infections (CLABSIs). The incidence of *S. haemolyticus*-associated CLABSIs in French neonates is increasing, but the mechanisms underlying this trend remain unclear.

**Methods:**

We examined microorganisms in 108 central line infusion sets used in preterm infants across 12 neonatal intensive care units, and collected at the time of removal.

**Results:**

The infusion sets varied widely in type (28 types; 1-6 parts) and length (10-180 cm, mean 52.9 cm). Contamination was detected in 24 infusion sets (22.2%), mainly by coagulase-negative *Staphylococci* (50.0%) and *Bacillus* species (41.7%). Higher contamination rates were linked to longer infusion lines (> 50 cm; *p* < 0.001), usage beyond 7 days (p = 0.002), and multi-line infusion systems (*p* < 0.001).

**Discussion:**

Our findings are fully consistent with guidelines, which recommend simpler designs and a 4 or 7-day use of infusion sets, emphasizing the importance of adhering to these guidelines to reduce the risk of CLABSIs. Additionally, our findings raise concerns regarding the use of multi-line infusion systems. These devices, which combine extended infusion line length, manufacturer-authorized use of up to 21 days, and intermittent use of certain infusion lines, are easily contaminated during use, creating a high-risk situation for central line contamination.

## 1 Introduction

The injectable route plays a crucial role in the care of low birth weight preterm newborns, particularly for the administration of continuous parenteral nutrition and medications. The exposure of these newborns to vascular catheterization is associated with an increased risk of central line-associated bloodstream infection (CLABSI), infections that contribute to higher morbidity, mortality, and increased hospital costs (Picaud et al., 2024). Multidrug-resistant coagulase-negative *Staphylococci* are well-known major causes of late-onset nosocomial CLABSIs among preterm newborns and represent a significant burden in neonatal intensive care units (NICUs) (Magnan et al., 2024). In France, the Surveillance and Prevention of Infections Associated with Invasive Devices (SPIADI) network has raised an alert since 2021 regarding the increasing incidence of late-onset *S. haemolyticus* CLABSIs in NICUs, particularly among preterm newborns characterized by a birth weight below 1,500 grams and a gestational age under 28 weeks (Spiadi, 2023). In a cohort of 14 NICUs, the incidence rate of *S. haemolyticus* CLABSIs increased from 1.90 per 1,000 catheter-days in 2020 to 4.31 in 2024, linked to the occurrence of clusters in several NICUs. Genomic studies on epidemic strains of *S. haemolyticus* ruled out the spread of a single clone (Magnan et al., 2024; Westberg et al., 2022; Martins-Simoes et al., 2024). However, the mechanisms driving the increasing incidence of *S. haemolyticus* CLABSIs in preterm newborns remain unclear.

*S. haemolyticus* is a notable component of the human skin microbiota and has a significant ability to survive in the environment and produce biofilm, two key characteristics that contribute to its involvement in late-onset CLABSI (Eltwisy et al., 2022). The connectors of infusion sets are critical entry points for microorganisms responsible for late-onset infections related to central venous catheters (Salzman and Rubin, 1997; Sitges-Serra et al., 1984). If strict aseptic techniques are not maintained during connections, disconnections, blood withdrawals, or pulse flushes performed on connectors, microorganisms can be introduced into the infusion line, colonize the device, and migrate endoluminally to the catheter. These conditions increase the risk of late-onset CLABSIs if the infusion sets near the central catheter are not regularly replaced. When infusions sets are left unchanged, microorganisms have enough time to migrate toward the catheter and contaminate it. For this reason, the Italian association of pediatric hematology and oncology for the management of the central venous access devices in pediatric patients with onco-hematological disease recommend to replace the infusion lines with a frequency of not more than 96 h (Cellini et al., 2022), and current guidelines recommend to replace the infusion lines with a frequency of at least every 7 days (World health organization, 2024; Société Française d’Hygiène Hospitalière, 2013). However, the evidence supporting these recommendations is not well-established, and the guidelines are not specifically neonatal guidelines. Recently, a manufacturer has validated their infusion sets for use up to 21 days, and encourages clinicians in neonatal intensive care units to use the system for a duration longer than that recommended by the current guidelines (Picaud et al., 2024).

For the past two decades, the care of premature newborns has evolved toward increasingly complex treatments. In these units, newborns are often exposed to several therapies where they may receive several intravenous drugs per day (Loureiro et al., 2019). These advancements have prompted manufacturers to develop innovative devices aimed at facilitating the administration of such treatments. Clinicians now have access to a wide variety of infusion systems, as well documented in Plaidy et al. (2022) study. The simultaneous infusion of multiple products implies the use of multi-lumen central venous catheters, peripherally inserted central catheters, or umbilical venous catheters, combined with multi-lumen extension lines and/or manifolds. As the number of treatments requiring infusion has increased, so has the frequency of line manipulations (e.g., connections, disconnections, blood withdrawals, or pulse rinses). Working inside the incubator is challenging for the HCWs, what makes it difficult to maintain the necessary aseptic conditions for line manipulations. Additionally, the growing number of connections exposed to the warm and humid environment of the incubator creates conditions that may promote bacterial biofilm formation on hubs and valves. To address these challenges, new devices have been recently developed to minimize the need for HCWs to intervene inside the incubator, near the central line insertion site (Van Rens et al., 2022). This is the case of multi-line infusion systems such as Edelvaiss® device. These systems first diminish the contact between medications infused in different infusion lines, and while it is a 95 cm-long line, it allows HCWs to perform most manipulations away from the catheter and outside the incubator.

There is a limited number of studies that have has yet investigated the impact of infusion set design and use duration on biofilm formation under real neonatal intensive care unit (NICU) conditions (Bayoumi et al., 2022). In the context of investigating the increased incidence of *S. haemolyticus*-associated CLABSIs in French neonates, we conducted a study aimed at detecting and characterizing microorganisms present in infusion lines from central venous catheters used in preterm infants and collected after their removal. The results were analyzed to explore factors that may contribute to endoluminal contamination of central infusion lines, particularly the potential role of infusion line design and replacement frequency in contamination by coagulase-negative *Staphylococci*.

## 2 Materials and methods

The study was proposed to all NICUs in France.

### 2.1 Line collection and transfer protocol

Infection control team leaders from the participating NICUs collected the lines used in their wards, following a standardized protocol, between March 1 and June 15. Below is an overview if the procedure.

#### 2.1.1 Collection process

Infusion lines were collected at the time of their removal, either when the infusion line was changed or when the central venous catheter was removed. To ensure the representativeness of the infusion lines used in the respective NICUs, the infusion lines were collected without specific selection. The collection was performed by an operator dressed in clean professional attire, including a hair cover, surgical mask, and sterile gloves. From the moment of removal, the infusion line was handled with sterile gauze impregnated with 70% alcohol and placed on a sterile drape.

#### 2.1.2 Preventing contamination of the infusion line’s interior during transfer

All hubs, valves, and infusion line extremities were disinfected with sterile gauze impregnated with 70% alcohol. Sterile caps were placed on each extremity without a valve. The conditioned infusion lines were then placed in sterile bags provided by the national team.

#### 2.1.3 Transfer to national laboratory

The infusion lines were promptly sent to the national laboratory, accompanied by a leaflet detailing a schematic representation of the infusion line, its duration of use, and whether it was used for blood transfusion, lipid infusion, or parenteral nutrition.

### 2.2 Microorganisms recovery

At the national laboratory, the infusion lines were subjected to a procedure based on a method originally used to detect microbial biofilm in flexible endoscope channels (Noubam-Tchatat et al., 2023).

#### 2.2.1 Validation process

Before applying this method to the infusion lines from the NICUs, we validated its effectiveness for biofilm detection. The procedure was tested on biofilms formed in sterile extension lines (V-green extension, 10 cm and 100 cm length, VYGON, Ecouen, France), similar to those used in NICUs. Nine test strains from the microbiology laboratory collection at the University Hospital of Tours, France, were used for validation (Supplementary Table S1). The extension lines were inoculated with 20 mL of 10–8 dilutions of each test strain suspension using a sterile syringe (Becton Dickinson, Madrid, Spain). The inoculated tubes, sealed at each end with sterile caps and enclosed in plastic bags, were incubated at 30°C or 37°C for three or 7 days, respectively. For microbial recovery, the tubes were flushed with 20 mL of 0.5% thiosulfate solution (Oxoid Deutschland, Wesel, Germany), the effluent was collected in sterile tubes, and filtered through a filtration manifold equipped with a fritted glass covered by a sterile 0.45 μm membrane (Millipore, Molsheim, France). The membrane was then placed on a Trypticase Soy Agar plate (Biomérieux, Marcy l’Étoile, France) and incubated for 3 days at 30°C or 37°C, depending on the biofilm formation temperature. Microbial detection and quantification were performed by counting colony-forming units on the agar plates. This validation was conducted twice to ensure reliability (Supplementary Table S2).

#### 2.2.2 Application to the infusion lines from the participating NICUs

Once validated, the procedure was applied to the infusion lines originating from the NICUs. Infusion lines were removed from plastic transport bags, placed under a microbiological safety hood on a sterile drape, and meticulously disinfected using sterile gauze soaked in 70% alcohol. The operator wore sterile gloves and handled the lines exclusively with sterile gauze. If the infusion line had separable parts, each part was treated individually. Microbial presence in each infusion line part was assessed as described above. All microbial colonies identified on the agar plates were characterized using MALDI-TOF technology (Bruker, Wissembourg, France). We defined major contamination as a microbial culture with 50 or more CFUs, and minor contamination as a culture with fewer CFUs. The microbiological results were analyzed in relation to the length of the infusion line, its duration of use, and its use for lipid infusion, blood transfusion, or parenteral nutrition.

### 2.3 Ethics approval and consent to participate

All experiments were performed in accordance with relevant guidelines and regulations. The study was carried out within the framework of the national agency Santé Publique France and the national program for the prevention of healthcare-associated infections (National Health Strategy 2023–2028), which advocates for regular evaluation of healthcare professionals’ practices by local infection control teams. All work related to this program was authorized by the CNIL (National Commission on Informatics and Liverties; file 2,212,596 dates March 27, 2019).

The protocol for the ChronoBIOline study was approved on Oktober 13, 2023, by an ethics committee/institutional review board during a meeting of the scientific council of Santé Publique France, which is responsible for overseeing the implementation of the SPIADI program. The implementation of the study was monitored by the scientific committee overseeing the SPIADI program (April 2, 2024). At the local level, participation in the study required written commitment from the hospital director and the infection control team leader. The commiment charters were collected at the national level by the SPIADI team. In the departments where the study was conducted, the observed professionals were informed about the study (observational component, microbiological component).

## 3 Results

Twelve NICUs (C1-C12), located in 10 university hospitals and two general hospitals across the country, participated in the study.

### 3.1 The infusion lines sent by the participating NICUs

In total, 108 infusion lines used with central venous catheters were sent to the national laboratory. The number of infusion lines sent by each center ranged from 3 to 18 (median: 8; IQR: 4.5). The infusion lines varied widely in design (Supplementary Table S3; Supplementary Figure S1). The most common infusion lines were two-part systems (50 lines, 46.3%) and single-part systems (40 lines, 37.0%). The remaining 18 systems were more complex, consisting of three to six parts. Twenty-one multi-line infusion systems were included in the study (19.4%), sourced from four of the 12 participating NICUs (C3, C4, C11, and C12; 33.3%). Filters were part of the infusion line systems in 11 cases (10.2%), coming from NICUs C6 and C10.

### 3.2 Microorganisms recovery

Depending on their design and the number of distinct accessible parts, between one and eight samples were collected per infusion line (Supplementary Table S4). In total, 309 samples were assessed for microbial cultures, resulting in positive cultures in 52 cases (16.8%).

#### 3.2.1 Contamination rate

Overall, 24 out of the 108 infusion lines studied (22.2%) were associated with at least one positive culture. The contamination rate was not significantly affected by the number of samples collected per infusion line (*p* = 0.089). Among the 12 NICUs, contamination rate for infusion lines ranged from 0 to 40.0%. However, differences in contamination rates between NICUs were not statistically significant, likely due to the limited number of infusion lines studied per NICU (*p* = 0.085).

#### 3.2.2 Microorganisms

A total of 29 different microorganisms were identified, as five of the 24 positive lines were contaminated by two different microorganisms each. The most common contaminants were coagulase-negative *Staphylococci* (12 infusion lines, 50.0%) and non-*cereus Bacillus* (10 lines, 41.7%). The *Staphylococci* identified in 12 infusion lines were of three species: *S. haemolyticus* (five infusion lines), *S. epidermidis* (five infusion lines), and *S. hominis* (two infusion lines). Major contamination was observed in eight out of these 12 cases (66.7%). The non-*cereus Bacillus* strains identified in 10 infusion lines belonged to 10 different species and were associated with minor contamination in all cases. Additionally, *Malassezia furfur* was associated with major contamination in two infusion lines from the same NICU (C6). *Micrococcus luteus* and *Paracoccus yeei* were each associated with minor contamination in one infusion line. Overall, major contaminations were more frequently associated with *Staphylococci* (8 out of 12) compared to other microorganisms (2 out of 12) (*p* = 0.038).

#### 3.2.3 Microbial recovery and design of central infusion lines

Among the 24 contaminated infusion lines, nine were one-part systems (37.5%), 14 were two-part systems (58.3%), and one was a more complex system (4.2%) (Supplementary Table S3). Overall, infusion line design (one-part, two-part, and three or more-part systems) did not influence contamination rates (*p* = 0.145; Table 1). In contrast, when considering the three types of infusion sets that each represented more than 10.0% of the studied sets (i.e., the 17 3-way extension lines, 16 Edelvaiss® systems, and the 12 3-way +1-way-extension lines), the contamination rate was higher for Edelvaiss® systems than for the other systems (50.0% for Edelvaiss® systems, vs. 0% for 3-way-extension lines, and 25.0% for 3-way +1-way-extension lines; *p* = 0.004). Similar results were observed when considering only staphylococcal contamination (*p* = 0.006), or *S. haemolyticus* contamination (*p* = 0.054).

**Table 1:** Contamination rates of the infusion sets according to design, infusion line length and duration of use.

Infusion sets		Contamination			Staphylococcal contamination			*S. haemolyticus* contamination		
	*N*	*N*	%	*P*	*N*	%	*P*	*N*	%	*P*
All	108	24	22.2		12	11.1		5	4.6	
According to design										
One-part systems	40	9	22.5	0.145	6	15.0	0.234	3	7.5	0.435
Two-part systems	50	14	28.0		6	12.0		2	4.0	
Three or more-part systems	18	1	5.6		0	0		0	0	
3-way extension line	17	0	0	0.004	0	0	0.006	0	0	0.054
Edelvaiss® system	16	8	50.0		5	31.2		3	18.7	
3-way +1-way extension line	12	3	25.0		0	0		0	0	
Multi-line systems	21	11	52.4	<0.001	7	33.3	0.001	4	19.0	0.003
Single-line systems	87	13	14.9		5	5.7		1	1.1	
According to infusion line length										
≤ 50 cm	59	6	10.2	<0.001	1	1.7	0.002	0	0	0.040
> 50 cm	49	18	36.7		11	22.4		5	10.2	
Multi-line systems	21	11	52.4	0.049	7	33.3	0.217	4	19.0	0.196
Single-line systems	28	7	25.0		4	14.3		1	3.6	
According to duration of use										
0–4 days	78	13	16.7	0.002	6	7.7	0.018	3	3.8	0.733
5–7 days	19	4	21.0		2	10.5		1	5.3	
> 7 days	11	7	63.6		4	36.4		1	9.1	
0–7 days	97	17	17.5	0.002	8	8.2	0.019	4	4.1	1
> 7 days	11	7	63.6		4	36.4		1	9.1	
0–4 days	78									
Multi-line systems	7	4	57.1	0.013	3	42.9	0.003	2	28.6	0.011
Single-line systems	71	9	12.7		3	4.2		1	1.4	
5–7 days	19									
Multi-line systems	5	2	40.0	0.567	1	20.0	1	1	20.0	0.580
Single-line systems	14	2	14.3		1	7.1		0	0	
0–7 days	97									
Multi-line systems	12	6	50.0	0.002	4	33.3	0.005	3	25.0	0.051
Single-line systems	85	11	12.9		4	4.7		1	1.2	
> 7 days	11									
Multi-line systems	9	5	55.6	0.712	3	33.3	1	1	11.1	1
Single-line systems	2	2	100		1	50.0		0	0	
Multi-line systems	21									
0–4 days	7	4	57.1	0.816	3	42.9	0.710	2	28.6	0.676
5–7 days	5	2	40.0		1	20.0		1	20.0	
> 7 days	9	5	55.6		3	33.3		1	11.1	
Single-line systems	87									
0–4 days	71	9	12.7	0.003	3	4.2	0.022	1	1.4	0.892
5–7 days	14	2	14.3		1	7.1		0	0	
> 7 days	2	2	100		1	50.0		0	0	

#### 3.2.4 Microbial recovery and length of central infusion lines

Infusion line lengths ranged from 10 to 180 cm, with a median length of 43.0 cm. One-part lines had the shortest median length at 16.0 cm (Supplementary Table S4). Among the 24 contaminated infusion lines, 18 were longer than 50 cm (75.0%). The median length of contaminated infusion lines was 95.0 cm, compared to 38.5 cm for non-contaminated infusion lines. Overall, infusion lines longer than 50 cm were more frequently contaminated than shorter infusion lines (36.7% vs. 10.2%; *p* < 0.001; Table 1). Similar findings were observed when considering only staphylococcal contamination (*p* = 0.002), or *S. haemolyticus* contamination (*p* = 0.040).

#### 3.2.5 Microbial recovery and duration of use before removal

The duration of use before removal was documented for all infusion lines, ranging from less than 1 day to 21 days (Supplementary Tables S5, S6). Of these, 78 were removed before the 5th day (72.2%), 19 between the 5th day and 7th days (17.6%), and 11 were removed after 7 days (10.2%). A significantly longer duration of use was observed with multi-line infusion systems (9/21; 42.9% used beyond 7 days vs. 2/87; 2.3% for single-line systems; *p* < 0.001), likely due to the manufacturer’s validation of Edelvaiss® system for up to 21 days. Contamination rates varied with duration of use: 16.7% for infusion lines removed before the 5th day, 17.5% for infusion lines removed before the 8th day, and 63.6% for infusion lines used beyond the recommended 7-day period (Table 1). Contamination rates were similar between infusion lines removed before the 5th day and those removed between the 5th and 7th days (13/78 vs. 4/19; *p* = 0.821). Overall, infusion lines removed before the 8th day were less frequently contaminated than those used beyond 7 days (*p* = 0.002). Among the infusion sets that were removed after 7 days of use, 10 were removed between the 8th and the 14th days, one was removed between the 15th day and the 21st day and none were removed after 21 days. Therefore, due to the small numbers of infusion sets, there were no significantly different contamination rates between the three groups (8–14; 15–21 and > 21 days).

#### 3.2.6 Microbial recovery and infusates

Among the infusion lines studied, seven were used for blood transfusion (6.5%), 83 for lipid infusion (76.8%), and 103 for parenteral nutrition (95.4%). The recovery of microorganisms was not influenced by the perfusion of infusate. Specifically, one infusion line among the 24 contaminated infusion lines was used for blood transfusion, compared to six out of the 84 non-contaminated infusion lines (*p* = 0.958); 19 infusion lines among the 24 contaminated infusion lines were used for lipid infusion, compared to 64 out of the 84 non-contaminated infusion lines (*p* = 0.976), and 23 infusion lines among the 24 contaminated infusion lines were used for parenteral nutrition, compared to 80 out of the 84 non-contaminated infusion lines (*p* = 1).

#### 3.2.7 Microbial recovery and multi-line infusion sets

We observed a higher contamination rate of multi-line systems (52.4%) compared to other infusions sets (14.9%; *p* < 0.001; Table 1). Similar result were found when considering only staphylococcal contamination (*p* = 0.001), or *S. haemolyticus* contamination (*p* = 0.003). To further investigate, we compared contamination rates in multi-line and single-line systems with similar lengths or durations of use. First, among the 49 infusion sets longer than 50 cm, contamination was higher in the multi-line systems (52.4%) than in the 28 single-line sets (25.0%; *p* = 0.049). Although not statistically significant, similar trends were observed for staphylococcal contamination (33.3% for multi-lines vs. 14.3% for single-lines), and *S. haemolyticus* contamination (19.0% for multi-lines vs. 3.6% for single-lines). Second, among infusion sets used less than 8 days, the contamination rate was higher in multi-lines (50.0%) than in the 85 single-line sets (12.9%; *p* = 0.002; Table 1). Similar results were observed when for staphylococcal contamination (*p* = 0.005), and *S. haemolyticus* contamination (*p* = 0.051). By contrast, there was no difference in contamination rates between multi-line and single-line systems used for more than 7 days (*p* = 0.712).

Overall, our data show an increased risk of infusion set contamination by microorganisms responsible for late-onset CLABSIs in premature neonates when infusion sets used with central venous catheters are long, used for more than 7 days, or include a multi-line system.

## 4 Discussion

Using a procedure adapted from one used to detect biofilm in flexible endocope channels, we investigated the presence of microorganisms in central infusion lines used for premature newborns in 12 NICUs. These 12 units represent one-sixth of all French NICUs.^1^ Therefore, we believe that our data provides a relatively accurate representation of the current situation in our country. In addition, with a high number of 108 infusion lines from central venous catheters examined, our experimental study provides valuable insights into microbial contamination in infusion lines used for the treatment and parenteral nutrition of infants.

We observed significant diversity in the central infusion lines used in NICUs, with considerable variation in design and length, which confirms previous findings (Plaidy et al., 2022). Despite guidelines recommending simpler systems (Société Française d’Hygiène Hospitalière, 2013; Société Française d’Hygiène Hospitalière, 2020; Haute Autorité de Santé, 2018), one in five infusion lines was very complex, consisting of at least three parts. One-third of the NICUs used multi-line infusion sets, and half of the infusion sets were longer than 50 cm. Based on the collective analysis of these data, we suggest that the frequent use of long, multi-access devices likely reflects the need for infusion sets that accommodate complex treatments, and the preference of HCWs to perform connector manipulations outside the incubator rather than inside, maintaining a relatively distant position from it. Another hypothesis is that longer infusion lines could facilitate the implementation of skin-to-skin care by parents, which is now commonly used for premature newborns.

In our study the use of long or multi-line systems was associated with high contamination rates of infusion sets. Using our procedure, microbial contamination was detected in one-quarter of the infusion lines, with heavy contamination in one out of 10 infusion lines studied. These findings are novel and represent a key aspect of our study. The microorganisms isolated from the studied infusion lines were primarily *S. haemolyticus* and *S. epidermidis*, which are microorganisms from human skin flora and are responsible for eight out of 10 neonatal late-onset CLABSIs in the national French database (Spiadi, 2023). The mechanisms of endoluminal contamination of infusion lines from central venous catheters are well-known (Mermel et al., 2009). Contamination primarily occurs during infusion set manipulations when aseptic conditions are not strictly maintained, following the contamination of a connector by microorganisms from the hands of HCWs, the patient’s skin, or the care environment. Finally, our results strongly suggest—though do not prove—that the most frequently observed endoluminal contaminations with the studied infusion lines may have predisposed the neonates in whom these infusion lines were used to the occurrence of late-onset CLABSIs associated with *S. epidermidis* and *S. haemolyticus*.

The second major group of microorganisms recovered from the infusion lines consisted of non-*cereus Bacillus*. Since *B. cereus* demonstrated the ability to produce biofilm under experimental conditions during the validation process, the possibility that this bacterium could develop biofilm following connector contamination should not be excluded. For non-*cereus Bacillus* strains, contamination was minimal in all but one case, which may reflect the difficulty of these environmental bacteria in colonizing infusion lines. These findings align with the limited role of these bacteria in causing neonatal CLABSIs, as indicated by their absence in our national survey since 2019 (Spiadi, 2023).

Infusion line length has long been considered a disadvantage due to the increased dead volumes associated with longer infusion lines, which can delay medication administration and increase the risk of precipitate formation from prolonged contact between different medications. Studies exploring infusion line length as a risk factor for infection are scarce (Bayoumi et al., 2022). Our findings, from both the experimental validation phase and the study of infusion lines used in NICUs, indicate that the contamination rate is significantly higher for longer infusion lines and suggest that infusion line length could facilitate the formation of biofilm inside the infusion lines. Longer infusion lines are favored for their ergonomic benefits, allowing easier handling outside the incubator and enhancing care comfort. A strict limitation on the use of infusion lines longer than 50 cm would conflict with HCWs’preferences for longer infusion lines. Consequently, while our results should not be used to ban the use of infusion lines longer than 50 cm, they should serve as a cautionary note for HCWs to carefully consider the infection risks associated with their use.

With two out of three infusion lines being removed or replaced before the 5th day of use, and nine out of 10 before the 8th day, it appears that practices generally align with the current guidelines (10;11;16;18). Although these guidelines lack specific evidence directly linking extended use beyond four or 7 days to increased infusion line contamination maximum of 4 days to limit, our study-while not designed to prove this-, provides data demonstrating that exceeding these durations is associated with a higher contamination rate. Our study does not argue for a change of the central catheters but recommends a change of the infusion sets linked to these catheters at a frequency of up to 7 days. We believe our findings can be used in educational sessions to raise HCWs’ awareness about the importance of adhering to recommended durations for central infusion line use. Considering these results, as well as those showing an increased risk of contamination for longer lines, we propose that infusion lines longer than 50 cm be used for a frequency of up to 4 days to limit the risk of contamination of the catheter to which they are connected.

At the very least, our data demonstrate a higher contamination rate with the multi-line infusion sets. These findings are logical, as these systems combine two risk factors for staphylococcal line contamination highlighted in our study: long infusion line length and frequent use beyond the maximum duration recommended by international guidelines. Furthermore, we suggest that for these infusion sets, the existence of multiple lines may promote frequent situations of intermittent infusion in one or more infusion lines of the system, which can then favor the development of biofilm if a microorganism has been introduced in the infusion lines. Overall, our data suggest that the use of the multi-line systems could promote CLABSIs in preterm neonates. This contrasts with a recent monocentric study, which reported that implementing this system in a NICU was followed by a reduction in neonatal CLABSIs (Picaud et al., 2024). In our study, contamination of the Edelvaiss® multi-line system was significantly associated with *S. haemolyticus*, a microorganism long linked to outbreaks in neonatal settings due to its particular ability to survive in the close environment of neonates (Martins-Simoes et al., 2024). Currently, as neonatal treatments encourage HCWs to use long infusion lines like the Edelvaiss® multi-line system to facilitate care, the incidence of *S. haemolyticus* is showing an increasing trend. This conjunction of factors suggests a possible link between the increasing complexity of treatments, the growing use of complex and long systems, and the rising occurrence of *S. haemolyticus* infections. Further studies should be conducted to test this hypothesis. While the results of our multicentric study should not be used to ban any innovative system, they should serve as a warning for clinicians to carefully consider the high infection risk associated with using these systems, particularly beyond the eighth day of use. Additionally, it serves as a reminder of the importance of maintaining strict aseptic conditions during all manipulations on the infusion set of central venous catheters, even those involving connectors far from the catheter. Future research should investigate whether shorter multi-lines, with strict adherence to the maximum recommended duration of use (e.g., up to 4 days, because of intermittent use of the infusion lines), along with the maintenance of rigorous aseptic techniques during manipulations, could mitigate the infection risk associated with these complex systems.

## 5 Limitations of the study

Our study has two major limitations. First, as a multicenter study based on very diverse practices (e.g., the use of various infusion sets with different infusion line length, numbers of catheter lumens, accesses, and practices), we did not investigate the impact of this diversity on the data obtained. Therefore, we cannot definitively exclude the possibility of center effects influencing the results. Second, the study design did not allow us to assess the impact of the endoluminal contamination detected in the infusion lines on the occurrence of CLABSIs in the preterm neonates who used the studied infusion sets. Further research is needed to link microbial recovery from an infusion set with systematic cultures of the catheter at removal and to examine clinical and biological signs of infection from the insertion of the central catheter up to 7 days after infusion line removal.

## 6 Conclusion

Our data provide evidence of high ability of staphylococci, particularly *S. haemolyticus*, to colonize infusion sets during their use. We identified the long length and duration of use of the infusion sets as risk factors for microbial contamination, supporting current guidelines that advocate for simpler infusion lines and a maximum duration of use not exceeding 7 days for the external infusion set (or 4 days in cases of intermittent use). Our findings do not endorse the use of long infusion sets or any usage beyond 7 days, and therefore do not support devices validated by manufacturers for use beyond this duration. We believe our work may contribute to improved adherence to existing recommendations regarding replacement of the external infusion sets.

Chiefly, our findings raise concerns regarding the use of multi-line systems. These devices, which combine extended infusion line length, manufacturer-authorized use of up to 21 days, and intermittent use of certain lines, are easily contaminated during use, creating a high-risk situation for catheter contamination. Given the severity of CLABSIs in preterm neonates and the side effects associated with the antibiotics typically used in these cases (primarily vancomycin and aminoglycosides), we believe our results should prompt clinicians to carefully consider the infection risk associated with the use of these systems. Moreover, as multi-line systems are increasingly used in human clinics, especially in oncology wards, we propose investigating the impact of these systems in those environments and closely monitoring the incidence rate of CLABSIs in patients using these devices.

## Data Availability

The original contributions presented in the study are included in the article/Supplementary material, further inquiries can be directed to the corresponding author/s.

## Footnotes

^1^12/67; https://data.drees.solidarites-sante.gouv.fr/explore/dataset/fichier_maternites_112021/information/.
