# Supplementary material for: Infusion line contamination in preterm neonates: impact of infusion line design, length, and use duration: the multicenter ChronoBIOline study
Source: Front Microbiol. 2025 Jan 24;15:1495568. doi: 10.3389/fmicb.2024.1495568 (PMC11802565; doi:10.3389/fmicb.2024.1495568)
Supplement: Supplementary file 1 [file Table_1.DOCX]

**Table 1.** Contamination rates of the infusion sets according to design, infusion line length and duration of use.

| **Infusion sets** | | **Contamination** | | | **Staphylococcal contamination** | | | ***S. haemolyticus* contamination** | | |
| --- | --- | --- | --- | --- | --- | --- | --- | --- | --- | --- |
|  | N | N | % | P | N | % | P | N | % | P |
| All | 108 | 24 | 22.2 |  | 12 | 11.1 |  | 5 | 4.6 |  |
| According to design | | | | | | | | | | |
| One-part systems | 40 | 9 | 22.5 | 0.145 | 6 | 15.0 | 0.234 | 3 | 7.5 | 0.435 |
| Two-part systems | 50 | 14 | 28.0 |  | 6 | 12.0 |  | 2 | 4.0 |  |
| Three or more-part systems | 18 | 1 | 5.6 |  | 0 | 0 |  | 0 | 0 |  |
|  | | | | | | | | | | |
| 3-way extension line | 17 | 0 | 0 | 0.004 | 0 | 0 | 0.006 | 0 | 0 | 0.054 |
| Edelvaiss® system | 16 | 8 | 50.0 |  | 5 | 31.2 |  | 3 | 18.7 |  |
| 3-way + 1-way extension line | 12 | 3 | 25.0 |  | 0 | 0 |  | 0 | 0 |  |
|  | | | | | | | | | | |
| Multi-line systems | 21 | 11 | 52.4 | <0.001 | 7 | 33.3 | 0.001 | 4 | 19.0 | 0.003 |
| Single-line systems | 87 | 13 | 14.9 |  | 5 | 5.7 |  | 1 | 1.1 |  |
| According to infusion line length | | | | | | | | | | |
| < 50 cm | 59 | 6 | 10.2 | <0.001 | 1 | 1.7 | 0.002 | 0 | 0 | 0.040 |
| > 50cm | 49 | 18 | 36.7 |  | 11 | 22.4 |  | 5 | 10.2 |  |
|  | | | | | | | | | | |
| Multi-line systems | 21 | 11 | 52.4 | 0.049 | 7 | 33.3 | 0.217 | 4 | 19.0 | 0.196 |
| Single-line systems | 28 | 7 | 25.0 |  | 4 | 14.3 |  | 1 | 3.6 |  |
| According to duration of use | | | | | | | | | | |
| 0-4 days | 78 | 13 | 16.7 | 0.002 | 6 | 7.7 | 0.018 | 3 | 3.8 | 0.733 |
| 5-7 days | 19 | 4 | 21.0 |  | 2 | 10.5 |  | 1 | 5.3 |  |
| > 7days | 11 | 7 | 63.6 |  | 4 | 36.4 |  | 1 | 9.1 |  |
|  |  |  |  |  |  |  |  |  |  |  |
| 0-7 days | 97 | 17 | 17.5 | 0.002 | 8 | 8.2 | 0.019 | 4 | 4.1 | 1 |
| > 7days | 11 | 7 | 63.6 |  | 4 | 36.4 |  | 1 | 9.1 |  |
|  |  |  |  |  |  |  |  |  |  |  |
| 0-4 days | 78 |  |  |  |  |  |  |  |  |  |
| Multi-line systems | 7 | 4 | 57.1 | 0.013 | 3 | 42.9 | 0.003 | 2 | 28.6 | 0.011 |
| Single-line systems | 71 | 9 | 12.7 |  | 3 | 4.2 |  | 1 | 1.4 |  |
|  |  |  |  |  |  |  |  |  |  |  |
| 5-7 days | 19 |  |  |  |  |  |  |  |  |  |
| Multi-line systems | 5 | 2 | 40.0 | 0.567 | 1 | 20.0 | 1 | 1 | 20.0 | 0.580 |
| Single-line systems | 14 | 2 | 14.3 |  | 1 | 7.1 |  | 0 | 0 |  |
|  |  |  |  |  |  |  |  |  |  |  |
| 0-7 days | 97 |  |  |  |  |  |  |  |  |  |
| Multi-line systems | 12 | 6 | 50.0 | 0.002 | 4 | 33.3 | 0.005 | 3 | 25.0 | 0.051 |
| Single-line systems | 85 | 11 | 12.9 |  | 4 | 4.7 |  | 1 | 1.2 |  |
|  |  |  |  |  |  |  |  |  |  |  |
| > 7days | 11 |  |  |  |  |  |  |  |  |  |
| Multi-line systems | 9 | 5 | 55.6 | 0.712 | 3 | 33.3 | 1 | 1 | 11.1 | 1 |
| Single-line systems | 2 | 2 | 100 |  | 1 | 50.0 |  | 0 | 0 |  |
|  |  |  |  |  |  |  |  |  |  |  |
| Multi-line systems | 21 |  |  |  |  |  |  |  |  |  |
| 0-4 days | 7 | 4 | 57.1 | 0.816 | 3 | 42.9 | 0.710 | 2 | 28.6 | 0.676 |
| 5-7 days | 5 | 2 | 40.0 |  | 1 | 20.0 |  | 1 | 20.0 |  |
| > 7days | 9 | 5 | 55.6 |  | 3 | 33.3 |  | 1 | 11.1 |  |
|  |  |  |  |  |  |  |  |  |  |  |
| Single-line systems | 87 |  |  |  |  |  |  |  |  |  |
| 0-4 days | 71 | 9 | 12.7 | 0.003 | 3 | 4.2 | 0.022 | 1 | 1.4 | 0.892 |
| 5-7 days | 14 | 2 | 14.3 |  | 1 | 7.1 |  | 0 | 0 |  |
| > 7days | 2 | 2 | 100 |  | 1 | 50.0 |  | 0 | 0 |  |
|  | | | | | | | | | | |
