# Supplementary material for: Infusion line contamination in preterm neonates: impact of infusion line design, length, and use duration: the multicenter ChronoBIOline study
Source: Front Microbiol. 2025 Jan 24;15:1495568. doi: 10.3389/fmicb.2024.1495568 (PMC11802565; doi:10.3389/fmicb.2024.1495568)
Supplement: Supplementary file 2 [file Table_2.DOCX]

**Supplementary table 1**. Bacterial test strains used in the *in vitro* study.

| Staphyloloccal test strains | Code | Biofilm production |
| --- | --- | --- |
| *S. aureus* ATCC 35556 | STA_AUR_1 | positive |
| *S. aureus* 2 | STA_AUR_2 | negative |
| *S. epidermidis CIP* 53124 | STA_EPI_1 | positive |
| *S. epidermidis*2 | STA_EPI_2 | positive |
| *S. capitis* | STA_CAP | negative |
| *S. haemolyticus* CIP 107204 | STA_HAE_1 | positive |
| *S. haemolyticus* 2 | STA_HAE_2 | positive |
| *Bacillus subtilis* ATCC 6633 | BAC_SUB | nk |
| *Bacillus cereus* CIP 5257 | BAC_CER | nk |

**Supplementary table 2.** Microbial recovery based on experimental conditions (tube length, temperature, and duration of exposure to 10^-8^ suspensions of test-strains).

We experimentally contaminated tubes of two different lengths (10 cm and 100 cm) by injecting 20 mL of a 10^-8^ dilution of an initial bacterial suspension of each test strain (Supplementary Table 1). The tubes were then incubated at either 30°C or 37°C for three or seven days. The results of these experiments are summarized in this table. Except for the *B. subtilis* test strain, which did not produce any biofilm under any conditions, the remainng eight strains were successfully recovered after the microbial recovery procedure. The highest recovery rate was observed for the 100 cm-long tubes, whereas, only three strains showed positive microbial recovery after three days in the 10 cm-long tubes.

**Supplementary Table 3**. Distribution of the infusion sets according to their design and the participating centers.

| Infusion sets | | | All | Participating centers | | | | | | | | | | | |
| --- | --- | --- | --- | --- | --- | --- | --- | --- | --- | --- | --- | --- | --- | --- | --- |
|  | |  |  | 1 | 2 | 3 | 4 | 5 | 6 | 7 | 8 | 9 | 10 | 11 | 12 |
| 1-part systems | | | 40 | 1 | 3 | 3 | 2 |  | 4 | 4 | 8 | 2 |  | 8 | 5 |
|  | 3-way extension line | | 17 |  | 3 |  |  |  | 4 |  | 8 |  |  |  | 2 |
|  | Edelvaiss® multi-line | | 16 |  |  | 3 | 2 |  |  |  |  |  |  | 8 | 3 |
|  | 4-way infusion manifold | | 3 |  |  |  |  |  |  | 3 |  |  |  |  |  |
|  | 4-way extension line | | 2 |  |  |  |  |  |  |  |  | 2 |  |  |  |
|  | 1-way extension line | | 1 | 1 |  |  |  |  |  |  |  |  |  |  |  |
|  | 2-way infusion manifold | | 1 |  |  |  |  |  |  | 1 |  |  |  |  |  |
| 2-part systems | | | 50 | 6 |  | 1 | 2 | 5 | 10 | 3 | 6 | 13 |  |  | 4 |
|  | 3-way- + 1-way-extension lines | | 12 |  |  |  |  | 5 | 6 |  |  |  |  |  | 1 |
|  | 4-way extension line + connector | | 7 |  |  |  |  |  |  |  |  | 7 |  |  |  |
|  | Two 3-way extension lines | | 7 |  |  |  |  |  |  |  | 6 |  |  |  | 1 |
|  | 2-way- + 4-way-extension lines | | 6 |  |  |  |  |  |  |  |  | 6 |  |  |  |
|  | 4-way infusion manifold + 1-way extension line | | 6 | 3 |  |  |  |  |  | 3 |  |  |  |  |  |
|  | 2-way- + 1-way-extension lines | | 3 |  |  |  |  |  | 3 |  |  |  |  |  |  |
|  | 3-way extension multi-line + connector | | 2 |  |  |  | 2 |  |  |  |  |  |  |  |  |
|  | 1-way extension line + connector | | 2 | 2 |  |  |  |  |  |  |  |  |  |  |  |
|  | Edelvaiss® multi-line + 1-way extension line | | 2 |  |  |  |  |  |  |  |  |  |  |  | 2 |
|  | 2-way extension multi-line + connector | | 1 |  |  | 1 |  |  |  |  |  |  |  |  |  |
|  | 2-way infusion manifold + 1-way extension line | | 1 | 1 |  |  |  |  |  |  |  |  |  |  |  |
|  | 2-way extension line + filter | | 1 |  |  |  |  |  | 1 |  |  |  |  |  |  |
| Complex systems with more than 2 parts | | | 18 | 1 |  | 1 |  | 2 |  |  | 4 |  | 10 |  |  |
|  | Two 3-way-extension lines + filter | | 7 |  |  |  |  |  |  |  |  |  | 7 |  |  |
|  | Three 3-way extension lines | | 3 |  |  |  |  |  |  |  | 3 |  |  |  |  |
|  | 1-way- + 2-way-extension lines + connector | | 1 |  |  | 1 |  |  |  |  |  |  |  |  |  |
|  | Two 1-way extension lines + connector | | 1 | 1 |  |  |  |  |  |  |  |  |  |  |  |
|  | 3-way- + two 2-way- + 1-way-extension lines | | 1 |  |  |  |  | 1 |  |  |  |  |  |  |  |
|  | Four 3-way extension lines | | 1 |  |  |  |  |  |  |  | 1 |  |  |  |  |
|  | Three 3-way extension lines + filter | | 1 |  |  |  |  |  |  |  |  |  | 1 |  |  |
|  | Three 3-way- + 1-way-extension lines | | 1 |  |  |  |  | 1 |  |  |  |  |  |  |  |
|  | Four 3-way extension lines + filter | | 1 |  |  |  |  |  |  |  |  |  | 1 |  |  |
|  | Five 3-way extension lines + filter | | 1 |  |  |  |  |  |  |  |  |  | 1 |  |  |
| Multi-line systems | | | 21 |  |  | 4 | 4 |  |  |  |  |  |  | 8 | 5 |
|  | | |  |  |  |  |  |  |  |  |  |  |  |  |  |
| All systems | | | 108 | 8 | 3 | 5 | 4 | 7 | 14 | 7 | 18 | 15 | 10 | 8 | 9 |
| Contaminated infusion lines | | | 24 | 1 |  | 2 | 2 | 2 | 3 | 1 | 1 | 4 | 1 | 5 | 2 |
| Contamination > 50 CFU | | | 10 |  |  | 1 | 1 |  | 3 |  |  | 2 |  | 1 | 2 |
| Contamination < 50 CFU | | | 14 | 1 |  | 1 | 1 | 2 |  | 1 | 1 | 2 | 1 | 4 |  |
| Non-contaminated infusion lines | | | 84 | 7 | 3 | 3 | 2 | 5 | 11 | 6 | 17 | 11 | 9 | 3 | 7 |

**Supplementary Table 4**. Distribution of the infusion sets by design and length.

| Infusion sets | | | All | Infusion line length (cm) | | |
| --- | --- | --- | --- | --- | --- | --- |
|  | |  |  | mean |  | median |
| 1-part systems | | | 40 | 47.5 |  | 16 |
|  | 3-way extension line | | 17 | 13.1 |  | 13 |
|  | Edelvaiss® multi-line | | 16 | 95 |  | 95 |
|  | 4-way infusion manifold | | 3 | 16 |  | 16 |
|  | 4-way extension line | | 2 | 34 |  | 34 |
|  | 1-way extension line | | 1 | 30 |  | 30 |
|  | 2-way infusion manifold | | 1 | 10 |  | 10 |
| 2-part systems | | | 50 | 53.7 |  | 55 |
|  | 3-way- + 1-way-extension lines | | 12 | 48 |  | 44 |
|  | 4-way extension line + connector | | 7 | 55 |  | 55 |
|  | Two 3-way extension lines | | 7 | 26.3 |  | 26 |
|  | 2-way- + 4-way-extension lines | | 6 | 63 |  | 63 |
|  | 4-way infusion manifold + 1-way extension line | | 6 | 36.3 |  | 36.5 |
|  | 2-way- + 1-way-extension lines | | 3 | 68 |  | 68 |
|  | 3-way extension multi-line + connector | | 2 | 130 |  | 130 |
|  | 1-way extension line + connector | | 2 | 30 |  | 30 |
|  | Edelvaiss® multi-line + 1-way extension line | | 2 | 108 |  | 108 |
|  | 2-way extension multi-line + connector | | 1 | 135 |  | 135 |
|  | 2-way infusion manifold + 1-way extension line | | 1 | 38 |  | 38 |
|  | 2-way extension line + filter | | 1 | 33 |  | 33 |
| Complex systems with more than 2 parts | | | 18 | 51 |  | 39 |
|  | Two 3-way-extension lines + filter | | 7 | 39;1 |  | 39 |
|  | Three 3-way extension lines | | 3 | 39 |  | 39 |
|  | 1-way- + 2-way-extension lines + connector | | 1 | 30 |  | 30 |
|  | Two 1-way extension lines + connector | | 1 | 180 |  | 180 |
|  | 3-way- + two 2-way- + 1-way-extension lines | | 1 | 43 |  | 43 |
|  | Four 3-way extension lines | | 1 | 52 |  | 52 |
|  | Three 3-way extension lines + filter | | 1 | 51 |  | 51 |
|  | Three 3-way- + 1-way-extension lines | | 1 | 43 |  | 43 |
|  | Four 3-way extension lines + filter | | 1 | 51 |  | 51 |
|  | Five 3-way extension lines + filter | | 1 | 77 |  | 77 |
| Multi-line systems | | | 21 | 101.5 |  | 95 |
|  | | |  |  |  |  |
| All systems | | | 108 | 52.9 |  | 43 |
| Contaminated infusion lines | | | 24 | 77.6 |  | 95 |
| Contamination > 50 CFU | | | 10 | 91.7 |  | 95 |
| Contamination < 50 CFU | | | 14 | 67.6 |  | 55 |
| Non-contaminated infusion lines | | | 84 | 45.9 |  | 38.5 |

**Supplementary Table 5**. Distribution of the infusion sets by duration between disposal and removal.

| Infusion sets | | | All | N infusion lines by duration between disposal and removal (days) | | |
| --- | --- | --- | --- | --- | --- | --- |
|  | |  |  | < 5 | 5 to 7 | > 7 |
| 1-part systems | | | 40 | 27 | 6 | 7 |
|  | 3-way extension line | | 17 | 14 | 3 |  |
|  | Edelvaiss® multi-line | | 16 | 6 | 3 | 7 |
|  | 4-way infusion manifold | | 3 | 3 |  |  |
|  | 4-way extension line | | 2 | 2 |  |  |
|  | 1-way extension line | | 1 | 1 |  |  |
|  | 2-way infusion manifold | | 1 | 1 |  |  |
| 2-part systems | | | 50 | 37 | 10 | 3 |
|  | 3-way- + 1-way-extension lines | | 12 | 10 | 2 |  |
|  | 4-way extension line + connector | | 7 | 6 | 1 |  |
|  | Two 3-way extension lines | | 7 | 7 |  |  |
|  | 2-way- + 4-way-extension lines | | 6 | 3 | 2 | 1 |
|  | 4-way infusion manifold + 1-way extension line | | 6 | 3 | 3 |  |
|  | 2-way- + 1-way-extension lines | | 3 | 3 |  |  |
|  | 3-way extension multi-line + connector | | 2 |  | 2 |  |
|  | 1-way extension line + connector | | 2 | 2 |  |  |
|  | Edelvaiss® multi-line + 1-way extension line | | 2 | 1 |  | 1 |
|  | 2-way extension multi-line + connector | | 1 |  |  |  |
|  | 2-way infusion manifold + 1-way extension line | | 1 | 1 |  |  |
|  | 2-way extension line + filter | | 1 | 1 |  |  |
| Complex systems with more than 2 parts | | | 18 | 14 | 3 | 1 |
|  | Two 3-way-extension lines + filter | | 7 | 4 | 2 | 1 |
|  | Three 3-way extension lines | | 3 | 3 |  |  |
|  | 1-way- + 2-way-extension lines + connector | | 1 | 1 |  |  |
|  | Two 1-way extension lines + connector | | 1 | 1 |  |  |
|  | 3-way- + two 2-way- + 1-way-extension lines | | 1 | 1 |  |  |
|  | Four 3-way extension lines | | 1 | 1 |  |  |
|  | Three 3-way extension lines + filter | | 1 |  | 1 |  |
|  | Three 3-way- + 1-way-extension lines | | 1 | 1 |  |  |
|  | Four 3-way extension lines + filter | | 1 | 1 |  |  |
|  | Five 3-way extension lines + filter | | 1 | 1 |  |  |
| Multi-line systems | | | 21 | 7 | 5 | 9 |
|  | | |  |  |  |  |
| All systems | | | 108 | 78 | 19 | 11 |
| Contaminated infusion lines | | | 24 | 13 | 4 | 7 |
| Contamination > 50 CFU | | | 10 | 5 | 2 | 3 |
| Contamination < 50 CFU | | | 14 | 8 | 2 | 4 |
| Non-contaminated infusion lines | | | 84 | 65 | 15 | 4 |

**Supplementary Table 6**. Data for all 108 infusion sets studied.
