# Supplementary material for: Infusion line contamination in preterm neonates: impact of infusion line design, length, and use duration: the multicenter ChronoBIOline study
Source: Front Microbiol. 2025 Jan 24;15:1495568. doi: 10.3389/fmicb.2024.1495568 (PMC11802565; doi:10.3389/fmicb.2024.1495568)
Supplement: Supplementary file 3 [file Table_3.DOCX]

**Supplementary Figure 1**. Photographs of the infusion sets observed at least three times among the 108 studied.

| **1-part systems** | |
| --- | --- |
| **Edelvaiss**® m**ulti-line system (C11/L1)** | **4-way infusion manifold (C7/L1)** |
| 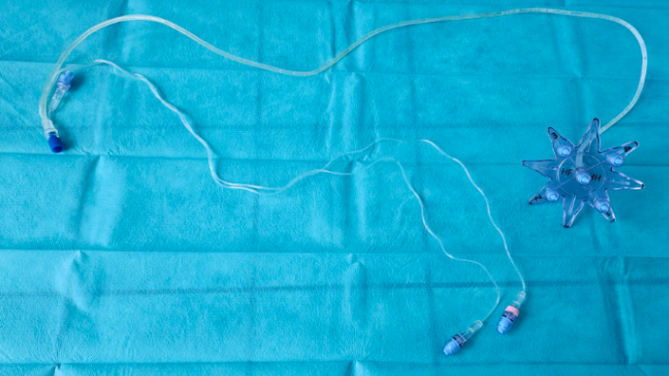  Line length : 95 cm | **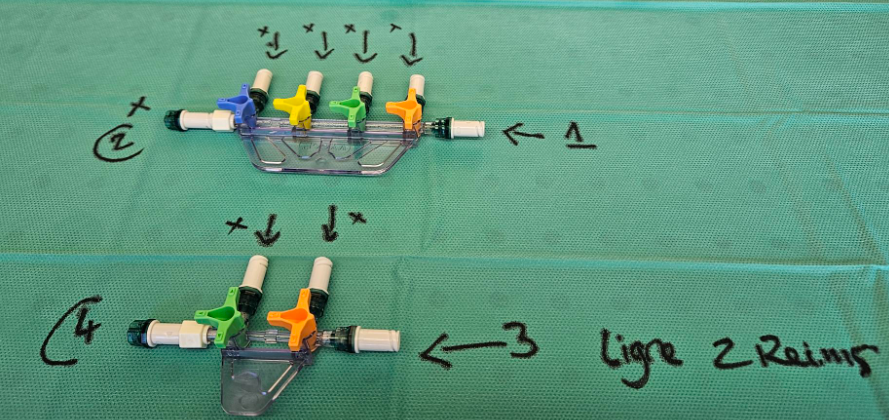**  Line length 16.0 cm  **3-way extension line (C8/L9)**  **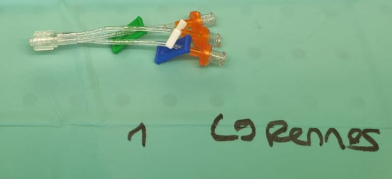**  Line length 13.0 cm |
|  |  |
| **2-part systems** | |
| **3-way- + 1-way-extension lines (C5/L3)** | **4-way extension line + connector (C9/L6)** |
| **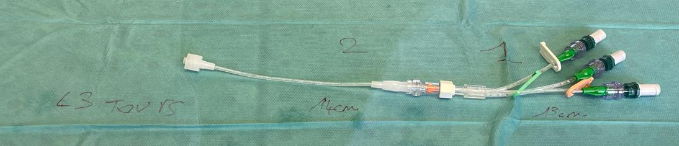** | **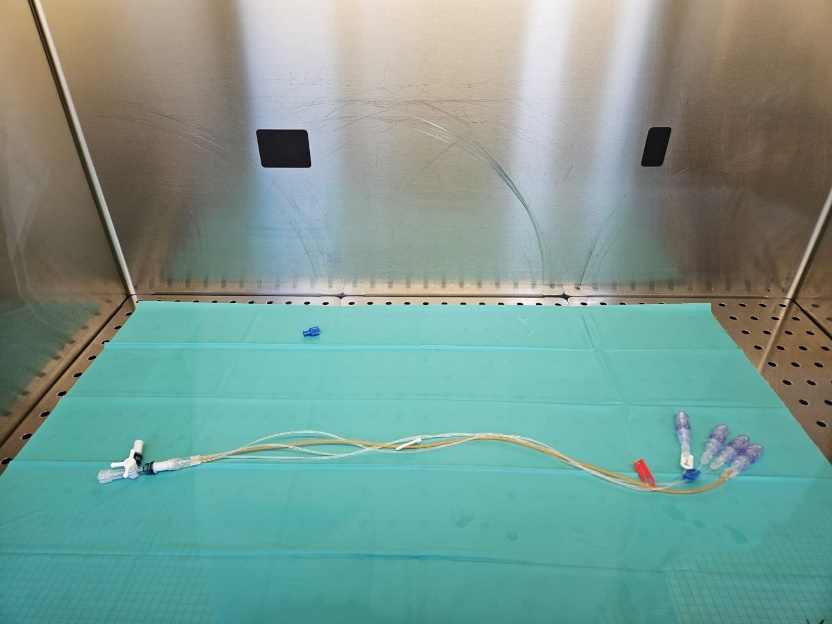** |
| Line length : 30.0 cm | Line length : 55.0 cm |
|  | |
| **Two 3-way extension lines (C8/L4)** |  |
| **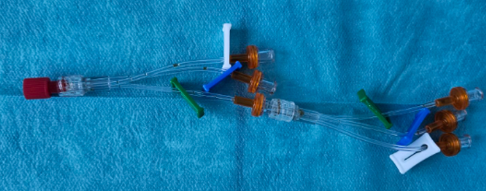** |  |
| Line length : 26.0 cm |  |
|  | |
| **4-way infusion manifold + 1-way extension line**  **(C7/L3)** | **2-way- + 4-way-extension lines (C9/L3)** |
| **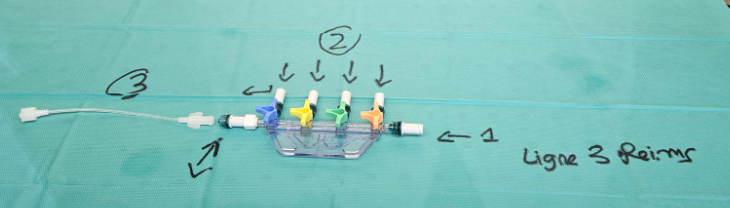** | 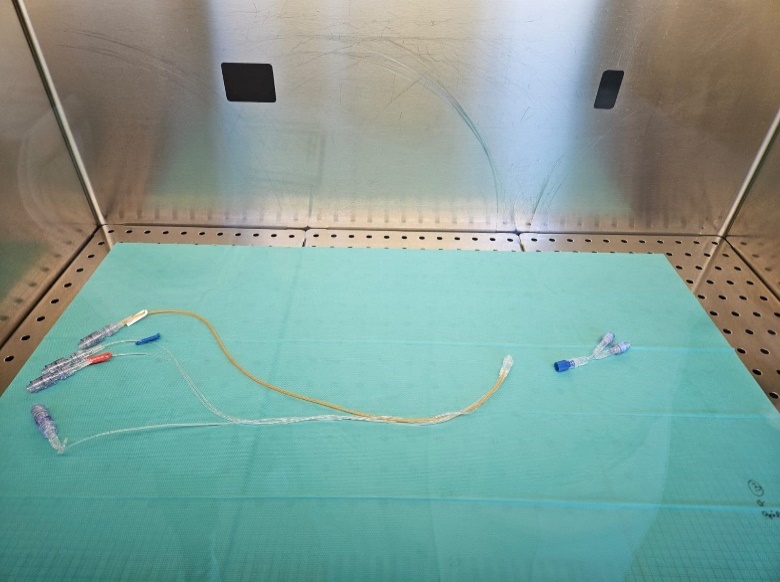 |
| Line length : 30.0 cm | Line length : 63.0 cm |
|  | |
|  | |
| **Complex systems with more than two parts** | |
| **Two 3-way extension lines + filter (C10/L3)** | **Three 3-way extension lines (C8/L12)** |
| **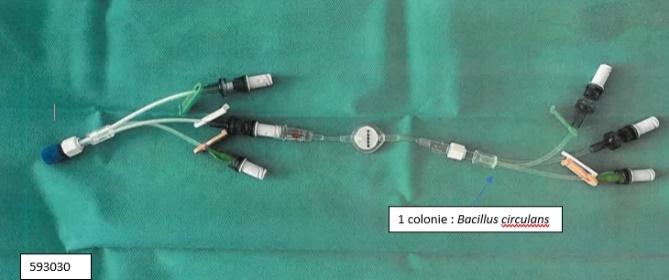** | **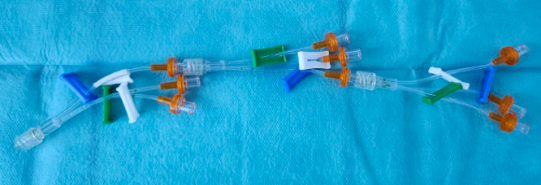** |
| Line length : 38.0 cm | Line length : 39.0 cm |
|  |  |
